# Ribosomal protein L22‐like1 promotes prostate cancer progression by activating PI3K/Akt/mTOR signalling pathway

**DOI:** 10.1111/jcmm.17663

**Published:** 2023-01-10

**Authors:** Xiaoyu Yi, Chao Zhang, Baojie Liu, Guojun Gao, Yaqi Tang, Yongzheng Lu, Zhifang Pan, Guohui Wang, Weiguo Feng

**Affiliations:** ^1^ School of Life Science and Technology Weifang Medical University Weifang China; ^2^ Department of Urology Surgery Shandong Cancer Hospital and Institute Jinan China; ^3^ Department of Urology Surgery Shandong First Medical University and Shandong Academy of Medical Sciences Jinan China; ^4^ Department of Urology Surgery Affiliated Hospital of Weifang Medical University Weifang China

**Keywords:** invasion, PI3K/Akt/mTOR, proliferation, prostate cancer, RPL22L1

## Abstract

Prostate cancer (PCa) is one of the most common malignancies in men. Ribosomal protein L22‐like1 (RPL22L1), a component of the ribosomal 60 S subunit, is associated with cancer progression, but the role and potential mechanism of RPL22L1 in PCa remain unclear. The aim of this study was to investigate the role of RPL22L1 in PCa progression and the mechanisms involved. Bioinformatics and immunohistochemistry analysis showed that the expression of RPL22L1 was significantly higher in PCa tissues than in normal prostate tissues. The cell function analysis revealed that RPL22L1 significantly promoted the proliferation, migration and invasion of PCa cells. The data of xenograft tumour assay suggested that the low expression of RPL22L1 inhibited the growth and invasion of PCa cells in vivo. Mechanistically, the results of Western blot proved that RPL22L1 activated PI3K/Akt/mTOR pathway in PCa cells. Additionally, LY294002, an inhibitor of PI3K/Akt pathway, was used to block this pathway. The results showed that LY294002 remarkably abrogated the oncogenic effect of RPL22L1 on PCa cell proliferation and invasion. Taken together, our study demonstrated that RPL22L1 is a key gene in PCa progression and promotes PCa cell proliferation and invasion via PI3K/Akt/mTOR pathway, thus potentially providing a new target for PCa therapy.

## 1 INTRODUCTION

Prostate cancer (PCa) is one of the most common malignant tumours in men, and its incidence continues to rise in many countries.^1^, ^2^, ^3^ It was estimated that 248,530 men were diagnosed and 34,130 men died from PCa in the United States in 2021.^4^ Currently, the early detection of PCa primarily relies on prostate‐specific antigen (PSA), but PSA testing has some flaws, such as the potential for overtreatment of some patients.^5^ Therefore, novel biomarkers need to be identified for PCa. Additionally, treatment of early PCa usually includes surgery and androgen deprivation therapy (ADT).^6^ However, nearly all PCa patients will eventually progress to castration‐resistant PCa (CRPC), with a median survival of no more than 2 years.^7^ Consequently, it is essential to further study the PCa pathogenesis and find new therapeutic targets.

Recently, ribosomal proteins were reported to be involved in cancer progression.^8^, ^9^ Ribosomal protein L22‐like1 (RPL22L1), a component of the 60S subunit, is regulated by RPL22.^10^ Several studies showed that RPL22L1 is involved in the regulation of cellular functions, including cell proliferation, migration, invasion and apoptosis, and is associated with ovarian cancer, colorectal cancer and hepatocellular carcinoma.^10^, ^11^, ^12^ In addition, Liang et al. found that RPL22L1 may be a promising biomarker for PCa.^13^ However, the role and potential mechanism of RPL22L1 in PCa remain unclear.

Various molecular mechanisms contribute to the progression of PCa, among which PI3K/Akt/mTOR pathway is one of the primary causes.^14^, ^15^ PI3K/Akt/mTOR pathway has been reported to be related to the formation and progression of PCa, biochemical recurrence after radical prostatectomy, and drug‐resistance.^16^, ^17^ Additionally, several studies found that this signalling pathway's inhibitor has the potential to be a therapeutic agent for hormone‐sensitive PCa and CRPC.^18^, ^19^, ^20^ Accordingly, it is necessary to further investigate the mechanism of PI3K/Akt/mTOR pathway in PCa progression, providing clinical benefit to patients with PCa.

The purpose of this study is to investigate the role of RPL22L1 in PCa progression and its potential mechanisms, thus providing a potential new target for PCa therapy.

## 2 MATERIALS AND METHODS

### 2.1 Reagents and antibodies

The tissue microarray was purchased from Alenabio Co. Ltd. Foetal bovine serum (FBS) and Dulbecco's Modified Eagle's Medium (DMEM) were obtained from Thermo Fisher Inc. Anti‐RPL22L1 and the secondary antibodies were purchased from Proteintech. CCK‐8 kit, transwell chambers, enhanced chemiluminescence reaction kit (ECL), bovine serum albumin (BSA), LY294002, anti‐PI3K (p85), anti‐p‐PI3K (p85), anti‐Akt, anti‐p‐Akt (Ser473), anti‐mTOR, anti‐p‐mTOR (Ser2448) and anti‐β‐actin were obtained from Beyotime Biotechnology. RPL22L1 lentiviral activation particles and RPL22L1 shRNA lentiviral particles were purchased from Santa Cruz.

### 2.2 Bioinformatics analysis

The gene expression profile (GSE55945) was analysed by GEO2R tool (https://www.ncbi.nlm.nih.gov/geo/geo2r/). The expression of RPL22L1 was verified by Oncomine (https://www.oncomine.org/) and UALCAN database (http://ualcan.path.uab.edu/). In addition, the effect of RPL22L1 expression on overall survival was tested by GEPIA database (http://gepia.cancer‐pku.cn/).

### 2.3 Cell culture and lentivirus transfection

The PCa cell lines (LNCaP, 22Rv1, DU145 and PC3) were purchased from Shanghai Cell Bank and cultured in DMEM supplemented with 10% FBS in a humidified atmosphere with 5% CO_2_ at 37°C. The stable cell lines shRPL22L1‐PC3 and overexpressed RPL22L1‐LNCaP were obtained by transfection of RPL22L1 shRNA lentiviral particles and RPL22L1 lentiviral activation particles, respectively.

### 2.4 CCK‐8 and cell colony formation

For the CCK‐8 assay, relative cell viability was monitored after seeding PC3 and LNCaP cells (2000 cells per well) in 96‐well plates, respectively, as described in our previous study.^21^ The values of light absorbance were tested by the microplate reader. For the assay of cell colony formation, as in previous studies,^22^ colony formation was detected after seeding PC3 and LNCaP cells (500 cells per well) in 6‐well plates for 14 d, respectively. Briefly, cell colonies were fixed with methanol for 20 min and stained with 0.1% crystal violet for 15 min. Finally, the cell colonies were counted under a microscope.

### 2.5 Wound‐healing and invasion

For the wound‐healing assay, PC3 cells were cultured in serum‐free medium, and then, a micropipette tip was used to create a cell‐free space as previously described.^23^ Photographs were taken with a microscope after 48 h. For the invasion assay, as described in the previous study,^24^ approximately 2.5 × 10^4^ PC3 and LNCaP cells were seeded separately into transwell chambers, and after 24 h of culture, the cells that passed through the membrane were stained with 0.5% crystal violet solution and counted.

### 2.6 Histopathology observation and immunohistochemistry

The experiments involving human tissue specimens in this study were in accordance with the Code of Ethics of the World Medical Association (Declaration of Helsinki). Histopathology observation was performed as previously described.^25^ Briefly, tissue samples were fixed in 10% neutral buffered formalin and then embedded in paraffin. Afterwards, the samples were cut to a thickness of approximately 4 μm and subjected to haematoxylin–eosin (HE) staining. Immunohistochemistry (IHC) was performed as described in our previous study.^26^ In short, tissue samples were treated with 3% H_2_O_2_ and followed by 10% BSA blocking. After incubating with anti‐RPL22L1 (1:250), anti‐p‐Akt (1:250), anti‐p‐mTOR (1:250) and secondary antibodies (1:2000), tissue samples were stained with 3,3‐diaminobenzidine for colour visualization and counterstained with haematoxylin.

### 2.7 Western blot

Western blot was carried out according to our previous reports.^27^ Briefly, proteins were subjected to SDS‐PAGE and then transferred to PVDF membranes. After incubating the membrane with primary antibodies, including anti‐RPL22L1 (1:1000), anti‐PI3K (1:1000), anti‐p‐PI3K (1:500), anti‐Akt (1:1000), anti‐p‐Akt (1:500), anti‐mTOR (1:1000) anti‐p‐mTOR (1:500), anti‐β‐actin (1:1000) and secondary antibodies (1:2000), the protein bands were detected with the ECL kit.

### 2.8 In vivo assay

In vivo assay was approved by the Animal Ethics Committee of Weifang Medical University. Five‐week‐old male nude mice (BALB/c) were divided into two groups and subcutaneously injected with control PC3 cells (5 × 10^6^ cells/mouse) and RPL22L1 low‐expressing PC3 cells (5 × 10^6^ cells/mouse), respectively. The growth of the tumour was measured every 1 week, and tumour volume was calculated using the following formula: volume (mm^3^) = 4π/3 × (width/2)^2^ × (length/2). After 5 weeks, mice were executed under deep anaesthesia. Tumours were collected, weighed and subjected to histopathology observation and IHC testing.

### 2.9 Statistical analysis

Data were analysed by anova or independent sample *t*‐test using SPSS 19.0 software. Experiments were repeated three times. Data were expressed as mean ± SD, and *p* < 0.05 represents statistical significance.

## 3 RESULTS

### 3.1 Identification of RPL22L1 by bioinformatics tool and IHC

**FIGURE 1:**
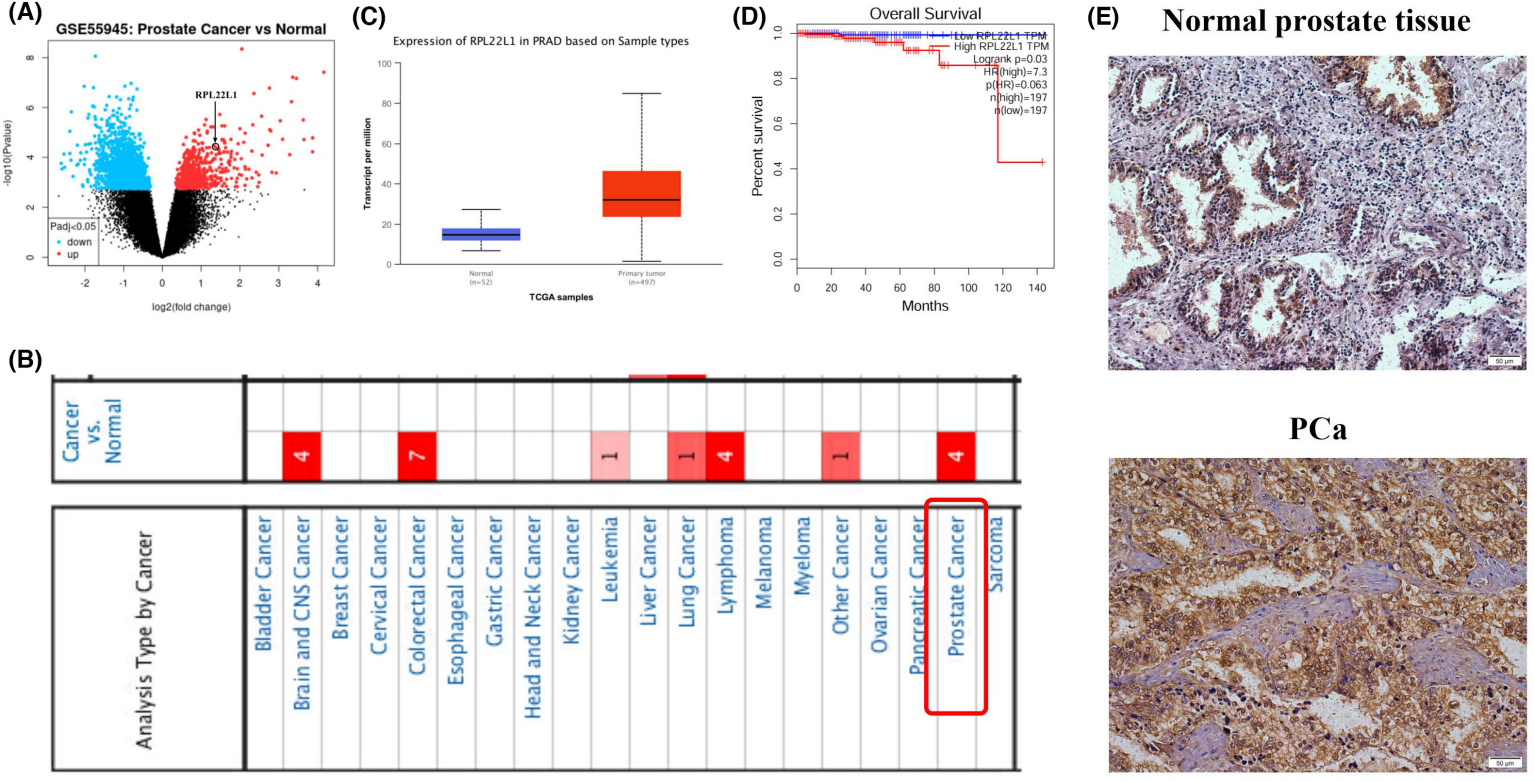
Identification of RPL22L1 by bioinformatics tool and IHC. (A) The gene expression profile (GSE55945) was analysed by GEO2R tool. (B) The expression of RPL22L1 from Oncomine database. (C) The expression of RPL22L1 from UALCAN database. (D) The effect of RPL22L1 expression on overall survival from GEPIA database. (E) The expression of RPL22L1 was validated by IHC. Scale bars, 50 μm.

The gene expression profile (GSE55945) was downloaded from GEO and analysed by GEO2R tool, which showed that RPL22L1 expression was significantly elevated in PCa tissues (Figure 1A). Subsequently, RPL22L1 expression was analysed by Oncomine, UALCAN and GEPIA database. The results showed that the expression of RPL22L1 was significantly higher in PCa tissue than in the normal prostate tissue (Figure 1B,C), and RPL22L1 expression was strongly associated with PCa prognosis (Figure 1D). In addition, RPL22L1 expression was further validated by IHC. The results suggested that RPL22L1 was localized in the nucleus and cytoplasm and was significantly more expressed in the PCa tissue compared with the normal prostate tissue (Figure 1E). The results above suggested that RPL22L1 may be a pivotal gene in the progression of PCa.

### 3.2 Establishment of RPL22L1 knockdown and overexpression cell lines

**FIGURE 2:**
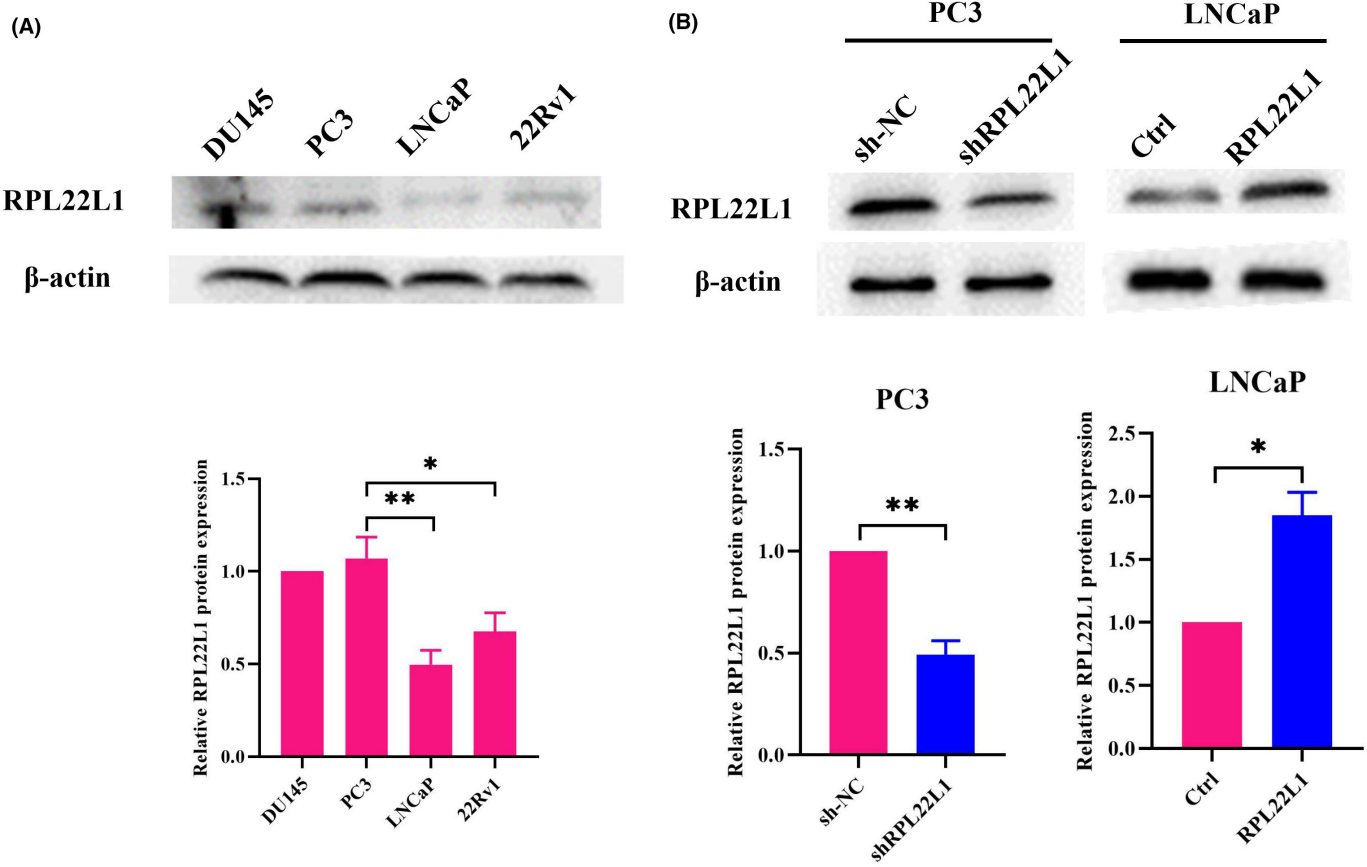
Establishment of RPL22L1 knockdown and overexpression cell lines. (A) The expression of RPL22L1 in different PCa cell lines. (B) The RPL22L1 knockdown and overexpression effects were verified by Western blot. The results are representative of three independent experiments. *p < 0.05, **p < 0.01. Error bars indicate SE.

The expression of RPL22L1 in different PCa cell lines was determined by Western blot. The results showed that RPL22L1 expression was highest in PC3 cells and lowest in LNCaP cells (Figure 2A). Therefore, PC3 cells were used to establish the RPL22L1 knockdown expression cell line, and LNCaP cells were used to establish the RPL22L1 overexpression cell line. As shown in Figure 2B, the expression of RPL22L1 in RPL22L1‐shRNA group was significantly lower than that in the control group, while the expression of RPL22L1 in RPL22L1‐overexpression group was significantly higher than that in the control group. These results proved that RPL22L1 knockdown and overexpression cell lines were successfully established.

### 3.3 RPL22L1 promotes PCa cells proliferation, migration and invasion

**FIGURE 3:**
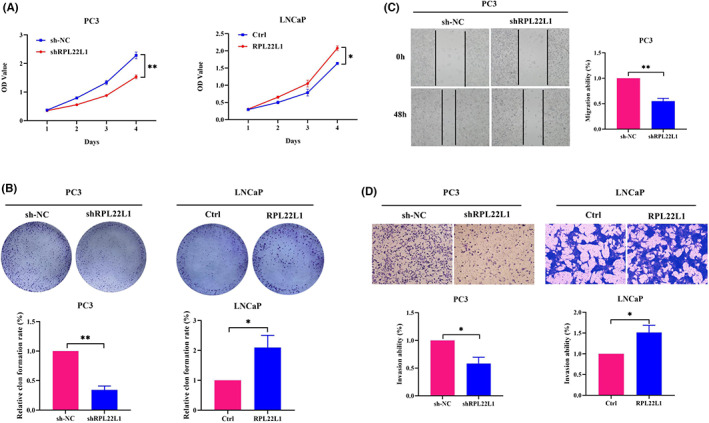
RPL22L1 promotes PCa cells proliferation, migration and invasion. (A) The effect of RPL22L1 on PCa cell proliferation was measured by CCK‐8 assays. (B) The effect of RPL22L1 on PCa cell proliferation was tested by colony formation assays. (C) The effect of RPL22L1 on PCa cell migration was assessed by wound‐healing assays. (D) The effect of RPL22L1 on PCa cell invasion was determined by transwell assays. The results are representative of three independent experiments. *p < 0.05, **p < 0.01. Error bars indicate SE.

To assess the role of RPL22L1 in PCa cells, CCK‐8, colony formation, wound‐healing and transwell assay were carried out. As shown in Figure 3A,B, knockdown of RPL22L1 significantly inhibited PC3 cell proliferation, whereas overexpression of RPL22L1 significantly promoted LNCaP cell proliferation (*p* < 0.05). Similarly, wound‐healing results suggested that knockdown of RPL22L1 significantly inhibited the migratory ability of PC3 cells (*p* < 0.05, Figure 3C). However, LNCaP cells adhered to each other and wound‐healing assay was difficult to perform, so the data for LNCaP were not shown. Transwell assay results proved that the invasion ability was remarkably suppressed by RPL22L1 knockdown, while significantly increased by RPL22L1 overexpression (*p* < 0.05, Figure 3D). In addition, apoptosis assay showed that knockdown of RPL22L1 elevated apoptosis in PC3 cells compared with the control (*p* < 0.05, Figure S1). Taken together, these results demonstrated that RPL22L1 promotes PCa cells proliferation, migration and invasion.

### 3.4 RPL22L1 activates PI3K/Akt/mTOR pathway in PCa cells

**FIGURE 4:**
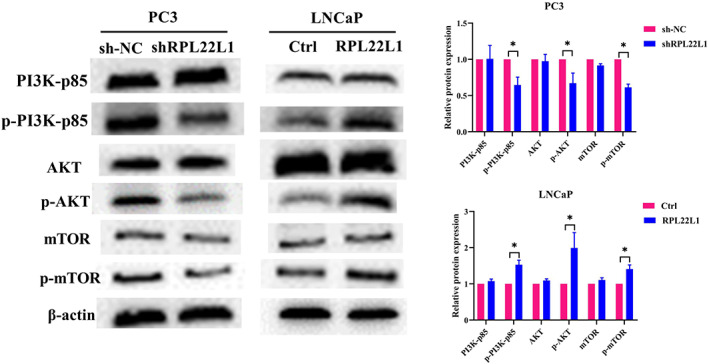
RPL22L1 activates PI3K/Akt/mTOR pathway in PCa cells. The expression and phosphorylation of the key genes of PI3K/Akt/mTOR pathway, including PI3K, Akt and mTOR, were tested by Western blot in PC3 and LNCaP cell lines. The results are representative of three independent experiments. *p < 0.05. Error bars indicate SE.

Given that PI3K/Akt/mTOR signalling pathway is closely related to PCa progression, we examined whether RPL22L1 activated PI3K/Akt/mTOR pathway in PCa cell lines. As shown in Figure 4, the expression of PI3K, Akt and mTOR was unchanged, but their phosphorylation level was decreased significantly in RPL22L1‐knockdown PC3 cells compared with the control group (*p* < 0.05). Conversely, in RPL22L1‐overexpressed LNCaP cells, the level of p‐PI3K, p‐Akt (Ser473) and p‐mTOR was elevated remarkably (*p* < 0.05). These data demonstrated that RPL22L1 activates PI3K/Akt/mTOR pathway in PCa cells.

### 3.5 RPL22L1 promotes PCa cell growth and invasion in vivo

**FIGURE 5:**
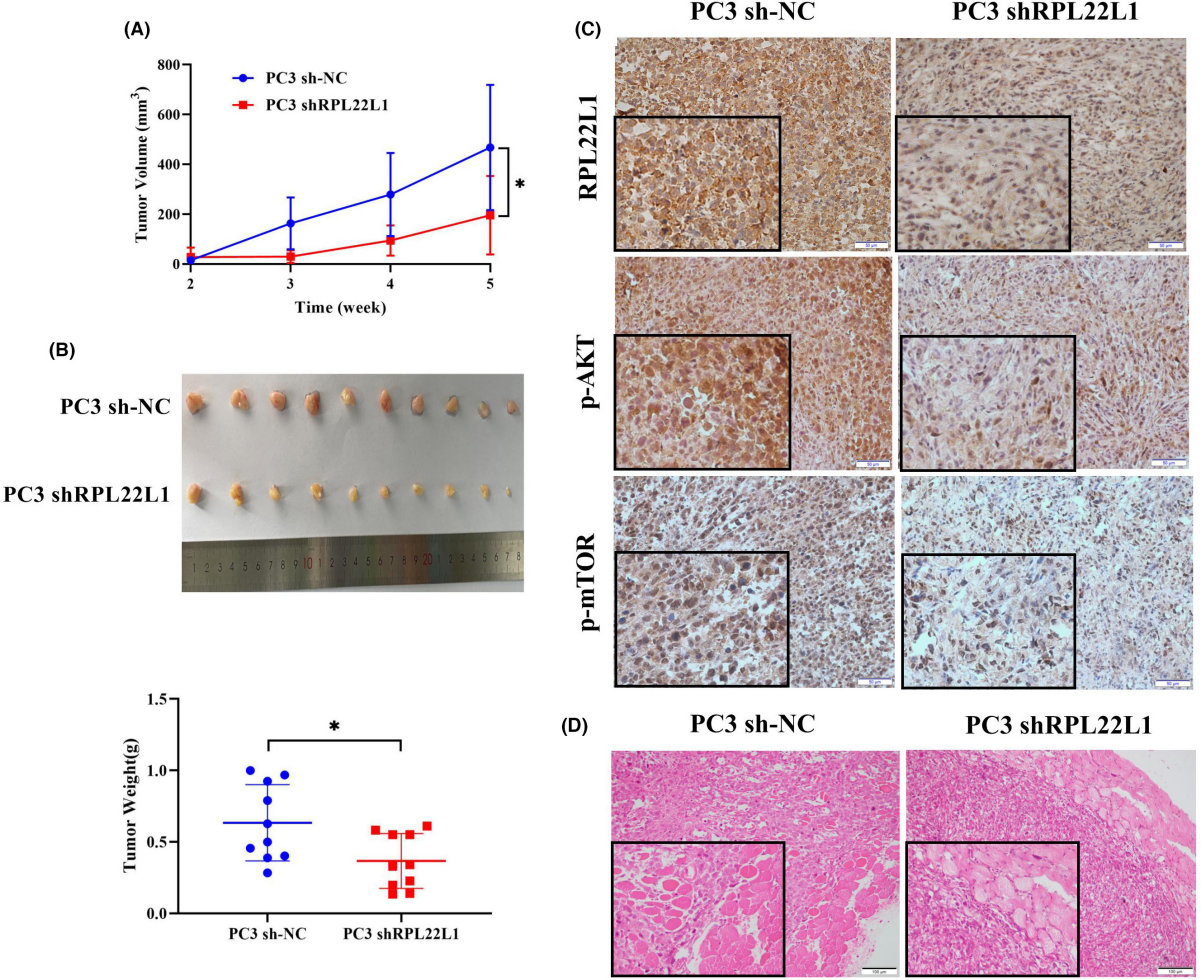
RPL22L1 promotes PCa cell growth and invasion in vivo. The sh‐NC and shRPL22L1 PC3 cells were injected subcutaneously into the nude mice, and tumour volume was examined at different time points. Finally, the nude mice were executed, the tumours were weighed, and the IHC and HE analysis were performed. (A) The tumour growth curve of nude mice derived from sh‐NC‐PC3 and shRPL22L1‐PC3. (B) The images and tumour weight of sh‐NC‐PC3 and shRPL22L1‐PC3 tumours were obtained on 5 weeks after tumour cell injection. (C) The expression of RPL22L1, p‐Akt, p‐mTOR in the tumour was detected by IHC. (D) HE staining of the tumour. *p < 0.05. Error bars indicate SE.

To further verify the effect of RPL22L1 on PCa cell growth and invasion in vivo, xenograft tumour assay was carried out. As shown in Figure 5A,B, PC3 cells with low RPL22L1 expression showed a weaker proliferative capacity. The tumour volume and weight were significantly decreased in the RPL22L1 low expression group compared to the control group (0.36 ± 0.19 vs. 0.63 ± 0.27 g, *p* < 0.05). IHC analysis revealed that the phosphorylation levels of Akt and mTOR were significantly lower in the RPL22L1 low expression group than the control group (Figure 5C). Additionally, histopathological analysis showed that tumour cells in the RPL22L1 low expression group had a weaker ability to invade into adjacent tissues compared with the control group, indicating that RPL22L1 enhanced PCa cell invasion (Figure 5D). To sum up, these data proved that RPL22L1 promotes PCa cell growth and invasion in vivo.

### 3.6 RPL22L1 contributes to PCa progression through PI3K/Akt/mTOR pathway

**FIGURE 6:**
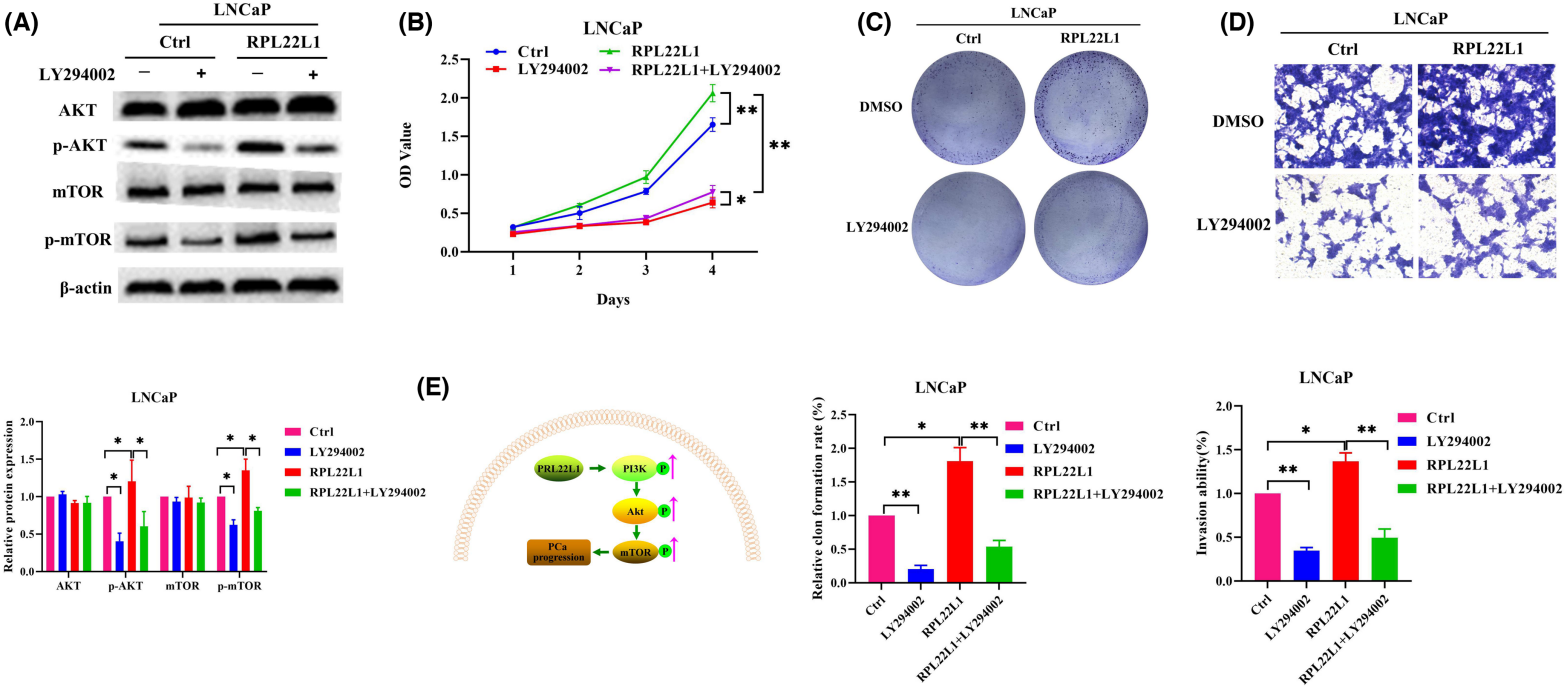
RPL22L1 contributes to PCa progression through PI3K/Akt/mTOR pathway. The PC3 cells in the control and RPL22L1 low expression groups were treated with or without LY294002 (25 μM) for 24 h. (A) The expression and phosphorylation of Akt and mTOR were tested by Western blot. (B, C) The cell proliferation was measured by CCK‐8 and colony formation assays. (D) The cell invasion was determined by transwell assays. (E) Proposed pathway involved in RPL22L1‐promoted PCa progression. The results are representative of three independent experiments. *p < 0.05, **p < 0.01. Error bars indicate SE.

To determine whether RPL22L1 exerts oncogenic effects in PCa through PI3K/Akt/mTOR pathway, LY294002, an inhibitor of PI3K/Akt pathway, was used to block this pathway. As shown in Figure 6A, LY294002 significantly suppressed the phosphorylation of Akt and mTOR promoted by RPL22L1 in LNCaP cells (*p* < 0.05). CCK‐8, colony formation and transwell analysis revealed that LY294002 remarkably abrogated the effect of RPL22L1 on LNCaP cell proliferation and invasion (Figure 6B–D; *p* < 0.05). Collectively, these results showed that RPL22L1 contributes to PCa progression through PI3K/Akt/mTOR pathway.

## 4 DISCUSSION

RPL22L1 has been suggested to play an essential role in the progression of various tumours.^10^, ^11^, ^12^, ^13^ However, its function and underlying molecular mechanism in PCa are still poorly understood. In this study, we found that RPL22L1 was significantly more expressed in PCa tissues than in normal prostate tissues. Further evidence from cell function experiments indicated that RPL22L1 significantly promoted PCa cell proliferation, migration and invasion. In addition, in vivo experiments showed that the low expression of RPL22L1 inhibited the growth and invasion of PCa cells in nude mice. Mechanistically, RPL22L1 activated PI3K/Akt/mTOR pathway, and blocking this pathway impaired the oncogenic function of RPL22L1 (Figure 6E).

In recent years, there is increasing evidence that the abnormal expression of ribosomal proteins is closely related to tumorigenesis.^8^, ^28^ Clarifying the role and mechanism of ribosomal proteins in tumour progression will provide new targets for tumour therapy. RPL22L1, a component of the ribosomal 60 S subunit, was determined for the first time in mouse brain tissue.^29^ Several studies in related fields have demonstrated that RPL22L1 is highly expressed in various tumours, including PCa,^13^ colorectal cancer^12^ and ovarian cancer.^11^ Likewise, our results showed that RPL22L1 expression was significantly upregulated in PCa and was strongly associated with patient prognosis, suggesting that RPL22L1 may be a pivotal gene in the PCa progression.

Currently, there are limited studies on the function of RPL22L1 in cancer progression. It has been reported that RPL22L1 promotes cell proliferation, migration and invasion in colorectal cancer^12^ and hepatocellular carcinoma.^10^ Liang et al. also found that the low expression of RPL22L1 inhibited PC3 cell proliferation and invasion in vitro.^13^ It should be noted that only PC3 cell was used to construct the low‐expressing cell line in their study, so the results need further confirmation. In the present study, PC3 and LNCaP cells were screened from four PCa cell lines to construct RPL22L1 low and overexpressed cell lines, respectively. Further cell functional experiments verified that RPL22L1 promoted PCa cell proliferation, migration and invasion. In addition, xenograft mice experiments confirmed that PRL22L1 promotes PCa cell growth and invasion in vivo. Collectively, our in vitro and in vivo results suggested that RPL22L1 plays a critical role in PCa progression.

PI3K/Akt/mTOR pathway is a critical carcinogenic pathway involved in tumorigenesis and progression in a wide variety of tumours, including PCa,^14^ non‐small cell lung cancer,^30^ breast cancer,^31^ lung cancer^32^ and colorectal cancer^33^ Previous studies have revealed that this pathway is associated with various cellular functions in PCa, such as cell proliferation, migration, invasion, apoptosis and autophagy.^15^, ^34^ Therefore, we hypothesized that RPL22L1 may promote PCa progression through PI3K/Akt/mTOR pathway. Our results suggested that RPL22L1 regulated the phosphorylation of several key molecules of PI3K/Akt/mTOR pathway, including p‐PI3K, p‐AKT and p‐mTOR. These results showed that RPL22L1 activated PI3K/Akt/mTOR pathway. Further results showed that LY294002, an inhibitor of PI3K/Akt pathway, was able to significantly attenuate the oncogenic effect of RPL22L1 on PCa cell proliferation and invasion, suggesting that RPL22L1 promoted PCa progression mainly by PI3K/Akt/mTOR pathway. In addition, the results showed that LY294002 could not completely abrogate the oncogenic effect of RPL22L1 on PCa, indicating that RPL22L1 may promote PCa progression through other mechanisms besides the PI3K/Akt/mTOR signalling pathway. Zhang et al. found that RPL22L1 activated ERK pathway to induce atypical epithelial‐to‐mesenchymal transition (EMT) in hepatocellular carcinoma.^10^ We speculate that ERK pathway may also be involved in PCa progression promoted by RPL22L1. Taken together, these results demonstrated that RPL22L1 contributes to PCa cell proliferation and invasion primarily by PI3K/Akt/mTOR pathway. However, it is not known how RPL22L1 regulates PI3K/Akt/mTOR pathway, which will be further investigated.

In conclusion, our study demonstrated that RPL22L1 is a key promoter of PCa and promotes PCa cell proliferation and invasion through PI3K/Akt/mTOR pathway, thus potentially providing a new target for PCa therapy.

## AUTHOR CONTRIBUTIONS

**Xiaoyu Yi:** Investigation (equal); methodology (equal). **Chao Zhang:** Data curation (equal); formal analysis (equal). **Baojie Liu:** Formal analysis (equal). **Guojun Gao:** Formal analysis (equal); methodology (equal). **Yaqi Tang:** Investigation (equal). **Yongzheng Lu:** Data curation (equal). **Zhifang Pan:** Conceptualization (equal); writing – review and editing (equal). **Guohui Wang:** Funding acquisition (equal); writing – review and editing (equal). **Weiguo Feng:** Conceptualization (equal); writing – original draft (equal).

## CONFLICT OF INTEREST

The authors confirm that there are no conflicts of interest.

## Supporting information


Figure S1

## Data Availability

The data that support the findings of this study are available on request from the corresponding author.
