# Supplementary material for: Ribosomal protein L22‐like1 promotes prostate cancer progression by activating PI3K/Akt/mTOR signalling pathway
Source: J Cell Mol Med. 2023 Jan 10;27(3):403–11. doi: 10.1111/jcmm.17663 (PMC9889667; doi:10.1111/jcmm.17663)
Supplement: Supplementary file 1 — Figure S1 [file JCMM-27-403-s001.zip › jcmm17663-sup-0002-Supplementary-Figure-legent-Fig. S1.docx]

**Figure legends**

**Fig. S1** The low expression of RPL22L1 promotes apoptosis in PC3 cells. The effect of RPL22L1 low expression on PC3 cell apoptosis was assessed by flow cytometric assay. The results are representative of three independent experiments. **P* < 0.05. Error bars indicate SE.
