# Supplementary figures and images for: Ribosomal protein L22‐like1 promotes prostate cancer progression by activating PI3K/Akt/mTOR signalling pathway
Source: J Cell Mol Med. 2023 Jan 10;27(3):403–11. doi: 10.1111/jcmm.17663 (PMC9889667; doi:10.1111/jcmm.17663)

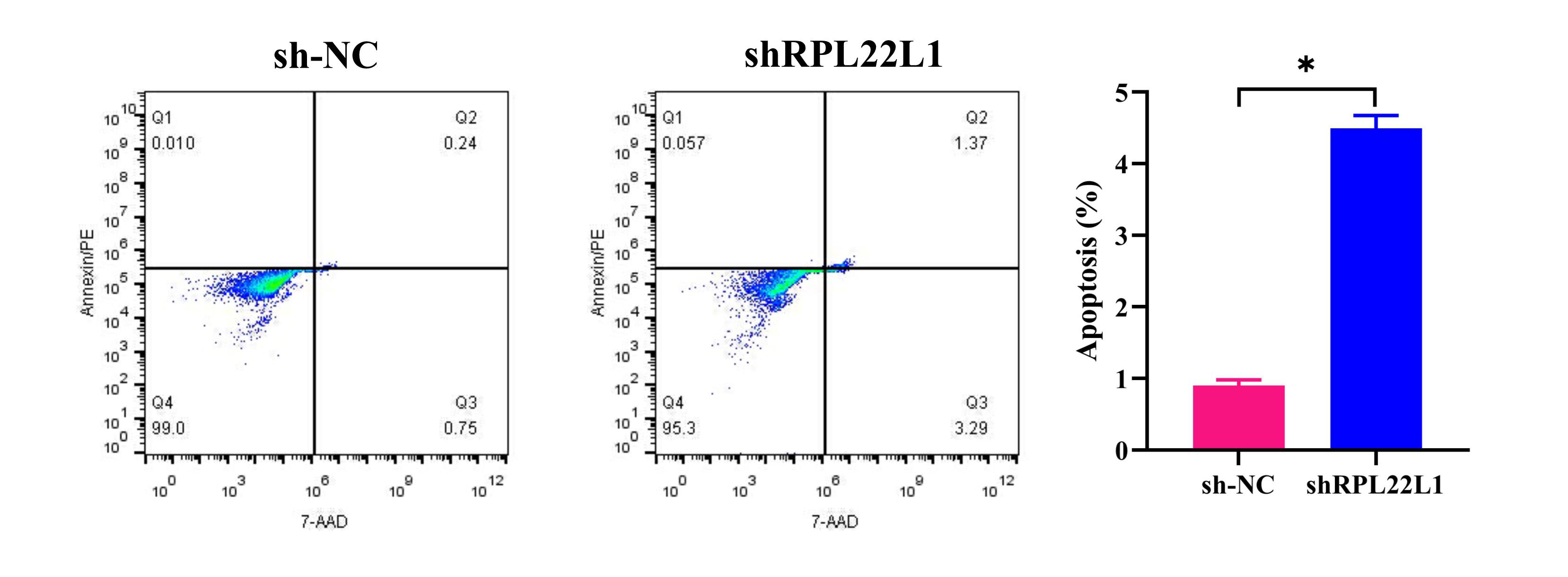

Supplement: Supplementary file 1 — Figure S1 [file JCMM-27-403-s001.zip › jcmm17663-sup-0001-Supplementary-Fig. S1.jpg]
